# Combination Chemotherapy in Severe Pulmonary Vein Stenosis—A Case Series

**DOI:** 10.3390/children10020364

**Published:** 2023-02-11

**Authors:** Gabriel Krivenko, Karen Iacono, David Nykanen, Robyn Keen, Robert Sutphin, Michael Farias

**Affiliations:** 1 School of Medicine, University of Central Florida, Orlando, FL 32837, USA; 2 The Heart Center, Orlando Health Arnold Palmer Hospital for Children, Orlando, FL 32806, USA; 3 Division of Hematology/Oncology, Orlando Health Arnold Palmer Hospital for Children, Orlando, FL 32806, USA; 4 Department of Cardiology, Boston Children’s Hospital, Boston, MA 02115, USA

**Keywords:** pulmonary vein stenosis, children, imatinib, sirolimus

## Abstract

Pulmonary vein stenosis results from a proliferative process that leads to the progressive obstruction of venous return to the left atrium. It is often resistant to catheterization and surgical based interventions and is frequently fatal when encountered in its severe form. Here, we describe three patients with severe, primary pulmonary vein stenosis that was progressing despite aggressive conventional management strategies. All three patients were initiated on combination chemotherapy with imatinib and sirolimus, drugs which have been previously shown to independently have potential benefit against PVS. Soon after the initiation of these therapies, all three patients experienced a stabilization of their disease process and clinical improvement. All three patients remain alive, with tolerable side effects from the medications. Although early in our experience and with only a small number of patients, combination chemotherapy with imatinib and sirolimus shows promise and merits further investigation as a therapeutic option for this aggressive disease.

## 1. Introduction

Pulmonary vein stenosis (PVS) is characterized by a proliferative process that can lead to the recurrent and relentless obstruction of venous return to the left atrium [1]. It is rare, affecting ~1.7 per 100,000 children under the age of two in its primary form and can also be secondarily associated with the surgical repair of anomalous pulmonary venous return [2]. Patients can present with a variety of symptoms, with the progressive nature of the disease leading to irreversible pulmonary vasculature remodeling, pulmonary hypertension, and ultimately death [3]. Interventional and surgical techniques to address this disease have poor durability [4,5,6,7], and the disease is often fatal with a reported 5-year mortality of 30–50% [8].

The pathophysiologic mechanisms underlying PVS remain unclear, with histological studies showing that it may be related to the uncontrolled proliferation of myofibroblast cells or their precursors [8,9]. Recent studies have shown the promise of adjunctive drug therapies targeted against myofibroblast proliferation, mainly imatinib and sirolimus, when combined with catheterization and surgical techniques [10,11]. Based on prior experience and an acceptable interaction profile, we decided to employ combination chemotherapy (CCT) with imatinib and sirolimus in three patients with severe, primary PVS. The patients were selected for this therapy because they were experiencing disease progression despite aggressive interventional therapy. Dosing was based on previously published regimens, and a multidisciplinary group from oncology, cardiology, and pharmacy created a treatment protocol with standardized follow-up and laboratory monitoring to titrate dosing and screen for side effects (see Supplementary Material File S1). The cases and our experience with this therapy are described here. Permission was obtained from the families of all three patients to report these deidentified cases, and this series was reviewed by our institutional review board and deemed exempt.

## 2. Cases

**Figure 1:**
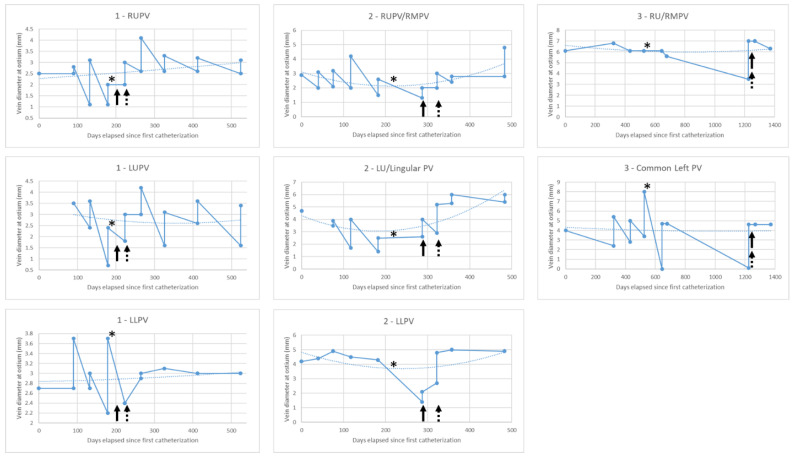
Temporal scatter plot of diseased pulmonary veins over time. The minimum osteal diameter of each of the eight diseased vessels among three patients was recorded based on angiography performed at catheterization. Day 0 was set as the date of the first catheterization where PVS was diagnosed. Any vertical jump represents an acute increase in diameter related to balloon dilation or stent placement. Though not detailed for each patient and each catheterization, our practice is to utilize the largest balloon that the distal vessel can accommodate in the dilation of a stenotic pulmonary vein and to eliminate any waist in the balloon; when a waist could not be eliminated, cutting balloon angioplasty was employed to facilitate dilation. Any fall in diameter over time represents disease recurrence or progression. A second-order polynomial trendline is displayed for each vessel. * = time of surgical intervention; solid black arrow = time of initiation of imatinib; dashed black arrow = time of initiation of sirolimus.

**Patient 1** was born prematurely at 26 weeks with a large, membranous ventricular septal defect (VSD) which required surgical closure at 4.5 months of age due to heart failure symptoms; the patient also required gastrostomy tube insertion due to poor feeding. At 6 months of age, narrowing of the pulmonary veins (PVs) was noted by echocardiography and by 9 months of age the patient required catheterization for PVS dilations in the context of worsening pulmonary hypertension (PHTN) as estimated by an echocardiogram. The patient had multi-vessel disease, involving the right upper (RU), left upper (LU), and left lower (LL) PVs, and had a calculated pulmonary vascular resistance (PVR) of 3.9 indexed Woods units. Despite balloon dilation, the disease recurred within weeks, leading to the near atresia of the RU and LU PVs, which required repeated interventions. At 12 months of age, the patient was referred to the operating room with plan to start chemotherapy with imatinib post-operatively due to the aggressive disease. The patient underwent sutureless repair of the RUPV, LUPV, and LLPV, with intraoperative stenting of the RUPV (4 mm diameter, 7.2 mm length) and LUPV (4 mm diameter, 4.8 mm length) utilizing cut, pre-mounted, and non-drug-eluting Palmaz Genesis stents (Cordis; Hialeah, FL, USA). Three weeks post-operation, after being cleared by cardiac surgery, the patient was started on imatinib 340 mg/m^2^ daily. According to an echocardiogram, the disease recurred within 6 weeks of surgery and the patient required repeat catheterization. Sirolimus was added at a dose of 1 mg/m^2^ daily, which was the dose required to maintain levels at the goal trough of 8–15 ng/mL. The patient has been maintained on CCT for over 10 months, requiring four additional catheterizations during this time for the recurrence of disease in the stented RUPV and LUPV; the LLPV disease has since stabilized, which we defined as no or minimal loss of vessel diameter in serial catheterizations. Importantly, the interval between required catheterizations increased from every 6–7 weeks to every 9–16 weeks (Figure 1; data available in Supplementary Material File S2). The patient has had two febrile, viral illnesses necessitating the temporary cessation of therapy and facial edema leading to an increased dose of furosemide on CCT; there have been no significant laboratory derangements noted on CCT.

**Patient 2** was born prematurely at 34 weeks and was diagnosed with trisomy 21, an atrial septal defect (ASD), a small muscular VSD, PHTN, and reflux with aspiration requiring gastrostomy tube insertion and Nissen fundoplication. Due to persistent oxygen requirement and the increased work required for breathing, the patient was referred for potential closure of the ASD at 2 months of age, but was discovered to have stenosis of the right sided PVs; the patient’s calculated PVR at the time was 3.8 indexed Woods units. The PHTN worsened with PVR rising to as high as 10 indexed Woods units, and the patient began catheterization interventions every 4–6 weeks on multi-vessel PVS involving the common RU/right middle (RM), common LU/lingular, and the LLPV. Atresia of the RUPV segment developed, and at 9 months of age the patient underwent sutureless repair of the RM and LU/lingular PVs with fenestrated ASD closure. The disease recurred, and 2 months post-operation, the patient required repeat catheterization. Because of disease recurrence, imatinib 340 mg/m^2^ daily was initiated. Five weeks later, after disease progression and repeat catheterization requirement, sirolimus 1 mg/m^2^ daily was added. The patient has been maintained on CCT for over 9 months, experiencing disease stabilization, the ability to dilate the veins with larger balloons during catheterization (Figure 1), and only two required catheterizations during this period. There was the occurrence of one febrile, viral illness requiring the temporary cessation of therapy, facial and extremity edema requiring increase in dose of furosemide, and one episode of thrombocytopenia with gastrostomy tube insertion-site bleeding related to supratherapeutic sirolimus levels, which improved after dose reduction. The final sirolimus dose required to maintain therapeutic trough was ~0.25 mg/m^2^ daily.

**Patient 3** was born prematurely as a twin at 25 weeks and was diagnosed with bronchopulmonary dysplasia and apical muscular VSDs, the latter of which resolved spontaneously. The patient lived abroad so care was coordinated internationally. PVS involving the common left PV was noted and was watched until a lung scan demonstrated a decrease in left lung perfusion to 21%; PVS was also noted in the RU/RMPV but was not intervened upon due to minimal narrowing and only a mild pressure gradient to the left atrium. At age 33 months of age, catheterization was performed to dilate the left PV, the result of which was not durable and the disease recurred. At age 40 months of age, catheterization for stenting of the left PV was complicated by stent malposition across the atrial septum; an operation was performed the following day to remove the stent and resect fibrous tissue from the common left and RU/RM PVs. At the 3-month post-operative follow-up, complete atresia of the left PV was noted and the patient developed hemoptysis. The atretic vein was successfully recanalized in the catheterization laboratory and stented with overlapping 5 mm Resolute Onyx zotarolimus-eluting stents (Medtronic; Minneapolis, MN, USA). Follow-up was delayed due to COVID-19 pandemic-related travel restrictions, and at the time of repeat catheterization over 18 months after recanalization, severe in-stent stenosis with near atresia of the vessel was noted. The vessel was re-opened and the stent dilated to 7 mm, although the distal vessel remained smaller; the RU/RMPV also required dilation for the first time. The patient was started on CCT with imatinib 340 mg/m^2^ daily and sirolimus 1 mg/m^2^ daily simultaneously, based on our experience with patients 1 and 2. Sirolimus was adjusted to ~0.8 mg/m^2^ daily to maintain therapeutic trough and CCT has continued for over 10 months. During this time the disease has been stable, with lung perfusion scans showing reduced but stable flow through the left lung and without required balloon dilations of the previously diseased vessels. The patient has had two febrile, likely viral, illnesses requiring the temporary cessation of CCT and did experience facial puffiness, subsequently requiring the initiation of furosemide therapy.

## 3. Discussion

PVS is a historically challenging disease with high mortality despite aggressive intervention. Its recognition as a proliferative process has permitted the examination of various medical targets against proliferation signaling pathways to combat this disease. While initial attempts to use chemotherapeutics such as vinblastine and methotrexate against PVS met with limited success [12], newer studies have shown improved outcomes when utilizing imatinib or sirolimus in addition to surgical and catheterization-based interventions. Imatinib mesylate (Gleevec, Novartis Inc; Basel, Switzerland) is a tyrosine kinase inhibitor that can affect the platelet-derived growth factor receptor and reduce myelofibroblast proliferation. A study from Boston Children’s Hospital showed that imatinib use, with or without the vascular endothelial growth factor receptor inhibitor bevacizumab, in 48 patients with severe PVS, led to a 72 week survival rate of 77% [10]. Sirolimus inhibits the mammalian target of rapamycin (mTOR) regulatory kinase. It, along with other mTOR inhibitors, has been shown to affect growth factor receptor pathways involved in myofibroblast proliferation and angiogenesis. A study from Children’s Healthcare of Atlanta showed that the use of sirolimus as a medical therapy for PVS in fifteen patients was associated with 100% survival at a median follow-up of 2.2 years [11].

As imatinib and sirolimus inhibit cellular proliferation through separate pathways, it is appealing to hypothesize that the two drugs in combination could have a synergistic effect. Indeed, vascular biology research on mouse-derived endothelial progenitor cells has shown such an effect of these medications through the synergistic enhancement of cell differentiation and apoptosis [13]. The simultaneous employment of systemic sirolimus in fourteen patients already on imatinib therapy for PVS was previously reported in a study on the prevention of in-stent stenosis; sirolimus courses were limited to ~8 weeks, and while no synergistic effect on the prevention of in-stent stenosis was noted, the drug combination seemed to be well tolerated by patients [14].

In this report, CCT with imatinib and sirolimus did appear to correlate with altered disease progression in three patients. As shown in Figure 1, the aggressive progression of PVS prompted consideration and the initiation of CCT, which was followed by the stabilization of the disease process in many of the affected vessels. Vessels that were becoming atretic or near-atretic remained patent, the recurrence of stenosis lessened, and intervals between required catheterizations increased. Vessel response was variable and, anecdotally, vessels that had undergone stent insertion responded less favorably to CCT than unstented vessels. We should note that in patients 1 and 2, imatinib was first initiated as monotherapy against PVS; sirolimus was added later, after both the publication of the Atlanta group’s study and the noting of disease recurrence on imatinib monotherapy. In patient 3, based on our positive experience with the first two patients, the medications were started simultaneously.

CCT has been tolerated in all three patients. It should be noted that imatinib is a moderate CYP3A4 inhibitor; therefore, a lower systemic sirolimus dose is required to achieve therapeutic goals. The maintenance dose of sirolimus in our three patient varied from 0.25-1 mg/m^2^ daily, emphasizing the importance of close laboratory value monitoring when titrating this regimen. One significant adverse event on CCT was the aforementioned thrombocytopenia and bleeding event in patient 2, related to supra-therapeutic sirolimus levels. In all three patients we noted increased edema, a well-described side effect of both medications, generally in the form of increased facial puffiness. We also observed transient and mild hypertriglyceridemia and hypercholesterolemia in all three patients, which improved without intervention. Lastly, and importantly, we did not observe any bacterial or serious infections in our patients on CCT, with immunosuppression and increased risk of infection being a prospective concern of ours; CCT was paused for any febrile illness experienced.

As with most studies on PVS, the evaluation of the isolated benefit of CCT in our series is limited by the multi-modal and variable interventions on the included patients—we were aggressive in surgical and catheterization interventions as the disease appeared to worsen, and decision regarding stenting was made on an individualized basis. Whether it is CCT or one of the drugs individually that is more helpful remains unknown, as does the optimal timing, dosing, and duration of these therapies. This emphasizes the importance of further study on these and other adjunctive medical therapies for PVS, including further basic science research. While we are happy to share our early and positive experience with CCT in a small number of patients, the further use of this therapy would be best approached through a well-designed prospective trial.

## Data Availability

The data presented in this study are available in the Supplemental Materials.

## Supplementary Materials

The following supporting information can be downloaded at: https://www.mdpi.com/article/10.3390/children10020364/s1, File S1: APH CCT Protocol; File S2: CCT De-identified data and graphs [10,11,14,15,16,17,18].

## Abbreviations

ASD = atrial septal defect; CCT = combination chemotherapy; LL = left lower; LU = left upper; mTOR = mammalian target of rapamycin; PHTN = pulmonary hypertension; PVR = pulmonary vascular resistance; PVS = pulmonary vein stenosis; PV = pulmonary vein; RL = right lower; RM = right middle; RU = right upper; VSD = ventricular septal defect.
