# Supplementary material for: Combination Chemotherapy in Severe Pulmonary Vein Stenosis—A Case Series
Source: Children (Basel). 2023 Feb 11;10(2):364. doi: 10.3390/children10020364 (PMC9954861; doi:10.3390/children10020364)
Supplement: Supplementary file 1 [file children-10-00364-s001.zip › File S1. APH CCT Protocol.pdf]

ARNOLD PALMER HOSPITAL
For Children

THE HEART CENTER

Overview of Gleevec® (Imatinib) and Sirolimus for Treatment of Intraluminal Pulmonary Vein Stenosis

Revised: July 29, 2021

Background

Intraluminal pulmonary vein stenosis (PVS) is a life-threating disease that leads to progressive obstruction of the pulmonary veins. The mechanisms underlying PVS are multifactorial and poorly understood, but previously studies have shown that some forms of PVS occur due to uncontrolled proliferation of myofibroblasts, cells that are typically involved in wound healing and contraction. Aggressive surgical and catheter-based options are often insufficient to treat PVS, leaving consideration for lung transplantation as a final surgical option.

In cases of severe PVS, defined as multi-vessel disease that is progressive despite interventional and/or surgical management, we propose novel and aggressive medical management with adjunctive chemotherapeutics that may provide benefit against this disease.

Gleevec® (imatinib mesylate) is a tyrosine kinase inhibitor that affects the PDGF Receptor, which has been shown to play a role in myofibroblast proliferation in PVS. Research from Boston Children's Hospital has shown promise that Gleevec, in conjunction with interventional and surgical management techniques, can help to promote survival in PVS. (1)

Sirolimus (Rapamune®) is an mTOR inhibitor which may also help inhibit myofibroblast proliferation. Research from Boston Children's Hospital and Emory University has shown that this medication can help slow the progression of myofibroblast and neo-intimal (within stent) proliferation, and improve outcomes in pulmonary vein stenosis. (2,3)

ARNOLD PALMER HOSPITAL
For Children

THE HEART CENTER

Arnold Palmer Hospital PVS Treatment Considerations

Inclusion Criteria:

- ≥ 4 weeks of age
- Severe PVS, defined as multi-vessel PVS that is progressive despite interventional or surgical management and/or is associated with severe pulmonary hypertension and RV dysfunction
- Laboratory criteria for initiation of medications:
    - CBC: Hgb > 10 g/dL, Platelets > 100,000, ANC > 1500
    - CMP: Cr < 1.5x ULN, ALT < 5x ULN, Bilirubin < 1.5x ULN
    - Sirolimus specific:
        - CMP: Albumin > 2 g/dL
        - Lipids: Fasting LDL < 160mg/dL, fasting triglycerides < 400mg/dL
        - UA: Proteinuria < 1+ of Urine Protein:Cr < 0.3
- ***Live attenuated vaccination should be held until discontinuation of medications***
    - ***If anticipating chemotherapy, can give accelerated vaccine schedule***
    - ***If vaccine needed, can pause for 2 weeks prior and 2 weeks post***

Exclusion Criteria:

- Concurrent severe medical disease, until controlled (infection, liver disease, kidney disease, diabetes, etc)
- Known HIV or immunodeficiency
- Females pregnant or breastfeeding
- *Sirolimus only*: Requirement for a strong CYP3A4 inhibitor such as some AEDs

THE HEART CENTER

Gleevec (Imatinib)

- Initial Imatinib dose 340mg/m2 daily, preferably with a meal
  - Consider adjunctive Ondansetron as needed for nausea/vomiting
  - See last page for compounding information
- Monitor after 2 weeks and then at least every other month:
  - CBC with differential
  - CMP (including LFTs)
- Common adverse reactions:
  - CV: Peripheral edema (~75%)
  - CNS: HA (~35%)
  - Derm (~10%): Eczema, skin rash, hypopigmentation
  - GI: GER (~25%), nausea/vomiting (~50%), diarrhea (~50%), abdominal pain (~40%)
  - NM: Myalgia (~50%), arthralgia (~30%)
  - Heme: Myelosuppression (~33%)
  - General: Weight gain (~25%)
- Holding parameters
  - Febrile illness (> 101.5F) or grade 4 infection
  - Cardiac surgery or procedure requiring wound healing (until recovered)
  - Significant laboratory derangements
    - ANC (= WBC x (% neutrophils + % bands)) < 750
    - Platelets < 75,000
    - Cr > 3x normal
- Dose adjustment: Only required if laboratory derangements are sustained for 2 weeks or more while holding imatinib, then reduce dose by 20% before resuming
- Duration: 48 weeks, or until 3 months (6 months if conservative) without disease progression

ARNOLD PALMER HOSPITAL
For Children

THE HEART CENTER

Drugs, food, and supplements that may interact with imatinib mesylate

<u>Antibiotics</u>
Clarithromycin, erythromycin, nafcillin, rifabutin, rifampin, rifapentin, telithromycin
<u>Antidepressants and antipsychotics</u>
Aripiprazole, brexpiprazole, nefazodone
<u>Antifungals</u>
Fluconazole, isavuconazole, itraconazole, ketoconazole, posaconazole, voriconazole
<u>Arthritis medications</u>
Leflunomide, tofacitinib
<u>Anti-rejection medications</u>
Cyclosporine, everolimus, tacrolimus, **sirolimus**
<u>Antiretrovirals and antivirals</u>
Asunaprevir, atazanavir, darunavir, delaviridine, efavirenz, etravirine, fosamprenavir, indinavir, lopinavir, nelfinavir, nevirapine, ritonavir, saquinavir, Stribild®, telaprevir
<u>Anti-seizure medications</u>
Carbamazepine, fosphenytoin, phenobarbital, phenytoin, primidone
<u>Pain medications</u>
Hydrocodone, oxycodone
<u>Heart medications</u>
Amiodarone, amlodipine, diltiazem, dronedarone, nilodipine, ranolazine, verapamil
<u>Other drugs</u>
Acetaminophen, apixaban, aprepitant, avanafil, bosentan, conivaptan, deferasirox, dexamethasone, eletriptan, eplerenone, ibuprofen, ivacaftor, lansoprazole, mifepristone, modafinil, natalizumab, netupitant, **sildenafil**, warfarin

<u>Food and supplements</u>
St. John's Wort
Grapefruit, grapefruit juice
Seville oranges
Star fruit

ARNOLD PALMER HOSPITAL
For Children

THE HEART CENTER

<u>Sirolimus</u>

- Initial Sirolimus dose 0.8mg/m2 two times daily, or 1.0mg/m2 daily if using in addition to imatinib (imatinib is a moderate CYP3A4 inhibitor and reliably increases concentrations of sirolimus)
  - No loading dose
  - Reduce dose by 33% for mild hepatic dysfunction
  - Target trough (drawn within 1h of next schedule dose): 8-15ng/mL
    - Obtain first trough 1 week after initiation or dose change
    - Repeat troughs weekly until within range x 2, then monthly
- Monitor at least monthly:
  - CBC with differential, CMP (including LFTs), Lipid profile, UA, sirolimus trough, blood pressure
- Initiation of PCP prophylaxis with either:
  - Sulfamethoxazole-trimethoprim
    - Infants/Children: 150mg TMP/m2/day given M/W/F (max 160mg TMP/day given M/W/F)
    - Adolescents: 160mg TMP given M/W/F
  - Pentamidine
    - IV 4mg/kg/dose every 3-4 weeks (max 300mg/dose)
- Common adverse reactions:
  - CV: Peripheral edema, HTN
  - CNS: HA, dizziness
  - Derm: Rash
  - Endo: Hypertriglyceridemia (~50%), hypercholesterolemia (~40%)
  - GI: Constipation (~35%), abdominal pain (~30%), diarrhea (~30%), nausea (~25%), mouth ulcers (~15%)
  - GU: UTI (~33%)
  - Heme: Anemia (~30%), thrombocytopenia (~25%)
  - NM: Arthralgia (~30%)
  - Renal: Increased creatinine (~40%)
- Holding parameters
  - Febrile illness (>101.5F) or grade 4 infection
  - Cardiac surgery or procedure requiring wound healing (until recovered)
  - Supratherapeutic level or significant laboratory derangements
- Dose adjustments: Depend upon toxicity and time until sirolimus level falls; consult with pharmacy
- Duration: Until at least 6 months without disease progression

ARNOLD PALMER HOSPITAL
For Children

THE HEART CENTER

Drugs, food, and supplements that may interact with sirolimus

Antibiotics
Erythromycin
Antifungals
Fluconazole
Posaconazole
Voriconazole
Heart medications
ACE inhibitors
Other drugs
CYP3A4 inducers or inhibitors (may anti-epileptic drugs)
Imatinib
Certain vaccines

Food and supplements
Grapefruit, grapefruit juice

ARNOLD PALMER HOSPITAL
For Children

THE HEART CENTER

References:

1. Callahan et al. Adjunct Targeted Biologic Inhibition Agents to Treat Aggressive Multivessel Intraluminal Pediatric Pulmonary Vein Stenosis. *J Pediatr*. 2018 Jul;198:29-35.e5.
2. Callahan et al. Systemic Sirolimus to Prevent In-Stent Stenosis in Pediatric Pulmonary Vein Stenosis. *Pediatr Cardiol*. 2020 Feb;41(2):282-289.
3. Patel et al. Systemic Sirolimus Therapy for Infants and Children With Pulmonary Vein Stenosis. *J Am Coll Cardiol*. 2021 Jun 8;77(22):2807-2818
4. www.UpToDate.com, sections on Imatinib, Sirolimus, Sulfamethoxazole-trimethoprim, and Pentamidine (accessed 6/30/2021)
5. Schultz KR, Bowman WP, Aledo A, et al. Improved early event-free survival with imatinib in Philadelphia chromosome-positive acute lymphoblastic leukemia: a children's oncology group study. *J Clin Oncol* 27:5175-81, 2009
6. Biondi A, Schrappe M, De Lorenzo P, et al. Imatinib after induction for treatment of children and adolescents with Philadelphia-chromosome-positive acute lymphoblastic leukaemia (EsPhALL): a randomised, open-label, intergroup study. *Lancet Oncol* 13:936-45, 2012
7. Chen H, Liu KY, Xu LP, et al. Administration of imatinib after allogeneic hematopoietic stem cell transplantation may improve disease-free survival for patients with Philadelphia chromosome-positive acute lymphoblastic leukemia. *J Hematol Oncol* 5:29, 2012

ARNOLD PALMER HOSPITAL
For Children

THE HEART CENTER

Orlando Health
Extemporaneous Preparations

Product	Imatinib 40 mg/mL in Ora-Sweet (Gleevec) **Hazardous Medication**
Concentration	40 mg/mL
Formulation	Oral Suspension

Ingredients

1. Imatinib 100 mg tablets — 40 tablets
2. Ora-Sweet (Paddock Perrigo) — QSAD: 100 mL

Compounding Instructions

The liquid formulation should be prepared in the pharmacy in a Biological Safety Cabinet as directed and dispensed according to Patient Care Policy #5090, *Hazardous Medications: Administration, Safe Handling and Disposal.*

1. Calculate the required quantity of each ingredient for total amount to be made.
2. Accurately weight and/or measure each ingredient.
3. Thoroughly pulverize the tablets with a glass mortar and pestle to a fine powder.
4. Geometrically incorporate sufficient Ora-Sweet with powder to final volume and mix well.
5. Package and label.

Storage Conditions Room temperature

Stability 14 Days

Auxiliary Labels

- For Oral Use Only
- Cytotoxic Agent- Dispose Properly
- Shake Well

Additional Notes **Hazardous Medication**
Chemotherapy agent

References

1.) Compounding today formula number 3536 *"Imatinib 40-mg/mL in Ora-Sweet [Paddock Perrigo]"*

Pharmacy Council approved November 2018
